# Numerical Simulation of Compressive Mechanical Properties of 3D Printed Lattice-Reinforced Cement-Based Composites Based on ABAQUS

**DOI:** 10.3390/ma17102370

**Published:** 2024-05-15

**Authors:** Weiguo Wu, Jing Qiao, Yuanyuan Wei, Wenfeng Hao, Can Tang

**Affiliations:** 1 Faculty of Civil Engineering and Mechanics, Jiangsu University, Zhenjiang 212013, China; 2 College of Mechanical Engineering, Yangzhou University, Yangzhou 225127, China; 3 College of Civil Science and Engineering, Yangzhou University, Yangzhou 225127, China

**Keywords:** 3D printing, cement-based composites, numerical simulation, mechanical properties

## Abstract

Research has established that the incorporation of 3D-printed lattice structures in cement substrates enhances the mechanical properties of cementitious materials. However, given that 3D-printing materials, notably polymers, exhibit varying degrees of mechanical performance under high-temperature conditions, their efficacy is compromised. Notably, at temperatures reaching 150 °C, these materials soften and lose their load-bearing capacity, necessitating further investigation into their compressive mechanical behavior in such environments. This study evaluates the compressibility of cement materials reinforced with lattice structures made from polyamide 6 (PA6) across different structural configurations and ambient temperatures, employing ABAQUS for simulation. Six distinct 3D-printed lattice designs with equivalent volume but varying configurations were tested under ambient temperatures of 20 °C, 50 °C, and 100 °C to assess their impact on compressive properties. The findings indicate that heightened ambient temperatures significantly diminish the reinforcing effect of 3D-printed materials on the properties of cement-based composites.

## 1. Introduction

The rapid expansion of the construction industry has led to the widespread adoption of concrete due to its exceptional compressive strength. Consequently, there has been a growing demand for enhancements in the material’s performance attributes. Concrete is recognized for its low tensile strength and susceptibility to brittle fractures, classifying it as a quasi-brittle composite material [1]. This vulnerability allows the formation of cracks under external stresses, facilitating the ingress of corrosive agents and thereby accelerating material degradation and diminishing structural durability. This phenomenon is particularly evident in traditional steel reinforcement, which is more likely to corrode under such conditions, significantly reducing the lifespan of structures. In response to these challenges, polymers fabricated through 3D-printing technology [2,3] have emerged as a novel solution, owing to their superior corrosion resistance compared to conventional rebar. This makes them capable of withstanding the penetration of harmful substances into the cement matrix. The advancement of 3D-printing technology has not only facilitated the creation of polymers with intricate lattice structures [4,5,6,7] but also holds the potential to expedite construction timelines and simplify the building process. As a result, 3D-printed polymer-lattice-reinforced cement-based composites have garnered substantial interest from the academic community, highlighting a promising direction for enhancing the durability and performance of concrete structures.

The integration of fiber materials into cement matrices has been demonstrated to effectively mitigate the brittleness inherent in cement-based materials. Notably, investigations into the enhancement of toughness through the inclusion of steel fibers have underscored the beneficial interactions between fibers and the cementitious matrix [8,9]. These interactions bolster the material’s energy absorption capacity via robust bonding, thereby augmenting the performance characteristics of cement. Nonetheless, achieving uniform dispersion of fibers within the cement presents significant construction challenges, revealing limitations in this approach. To address these limitations, Nam et al. [10] leveraged 3D-printing technology to design models capable of precisely controlling the orientation and placement of fibers in concrete. Their methodology involved examining the flexural mechanics of fiber-reinforced cement mortars with diverse spatial distributions through three-point bending tests. The advancements in 3D-printing technology have since captivated the research community, prompting extensive experimentation to explore the reinforcing potential of 3D-printed structures as alternatives to fiber materials in cement-based composites. Researchers like Farina [11] have prepared polymer and metal-reinforced 3D-printed structures with varied surface textures, employing them to fortify cement mortar. These studies, which included three-point bending tests, delved into the influence of surface roughness on the mechanical behaviors of cement mortars, revealing how steel bar surface roughness can amplify load-bearing capabilities by altering the adhesion with the cement mortar. Tian et al. [12] conducted experimental assessments on the uniaxial compressive strength of an innovative composite column comprising precast grid-reinforced ultra-high-performance concrete (UHPC) within stay-in-place formwork and post-cast concrete. The findings indicate that polymer grids with carbon fiber reinforcement (CFRP) can foster strain hardening and enhance ductility and toughness through improved lateral confinement compared to stainless steel (SS) grids. Rosewitz et al. [13] designed a 3D-printed biomimetic polymer, utilizing it to reinforce cement-based composites and analyze their mechanical properties and failure mechanisms across different geometric structures. Similarly, Xu et al. [14] and Salazar et al. [15] experimented with varying tactics of 3D printing to reinforce cementitious materials with polymer network structures and investigated the effects on the materials’ properties. Particularly, Salazar et al. [15] focused on ultra-high-performance concrete (UHPC) materials reinforced by 3D-printed polymer lattices, assessing their flexural behavior through four-point bending tests. These studies have collectively reinforced the notion that 3D-printed lattice structures can significantly improve the ductility of ultra-high-performance concrete materials. However, the existing research has mainly discussed the mechanical properties of 3D-printed structural reinforcement materials at room temperature, and paid little attention to the effect of high-temperature environment on 3d printed lattice-reinforced cement-based composites. Notably, the compressive mechanical properties of PA6 [16,17] lattice structures fabricated using Multi Jet Fusion (MJF) technology [18,19] degrade at elevated temperatures, potentially leading to softening of the polymer material. Herein, we assess the elastic modulus of PA6 under varying temperatures through experimental samples. The investigation reveals that high temperatures detrimentally affect the material’s elastic modulus. Thus, understanding the mechanical behavior of 3D-printed lattice-reinforced cement-based composites in high-temperature conditions is crucial. This study innovatively proposed the influence of a high-temperature environment on the mechanics of 3D-printed lattice-reinforced cement-based materials. Through numerical simulation, a high-temperature uniaxial compression test was carried out on 3D printed lattice-reinforced cement-based composite materials prepared by MJF technology, and its compression mechanical properties and failure mechanism were discussed.

## 2. Finite Element Model Modeling

### 2.1. Material Parameters

**Figure 1:**
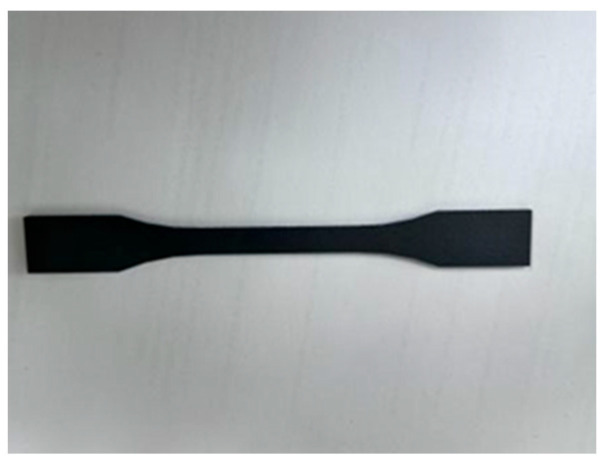
PA6 Elastic modulus measurement sample.

In this research, the specimens utilized were subjected to uniaxial compression testing. The material properties of the cement matrix are detailed in Table 1, which include a density of 2400 kg/m^3^, an elastic modulus of 30 GPa, and a Poisson’s ratio of 0.2. Additionally, the dilatancy angle is set at 30°, eccentricity at 0.1, the ratio of biaxial to uniaxial compressive strength at 1.16, and the tension-compression strength ratio (k) at 0.6667. The viscosity parameter is specified as 0.0005, with the failure mode characterized by the concrete damage plasticity (CDP) model [20,21,22,23]. The specimens were designed as cubic structures with a side length of 42 mm, featuring an internal lattice prepared using MJF technology. MJF employs powder materials and utilizes a binding agent and detailing agent, sprayed onto the powder; subsequently, energy is applied to fuse the material in the designated area. This 3D-printing technique is noted for producing samples whose mechanical properties are largely invariant to the build direction. Material parameters of the 3D-printed lattice, detailed in Table 2 and Table 3, indicate a mass density of 1100 kg/m^3^ and a Poisson’s ratio of 0.38. To accurately assess the impact of high-temperature environments on the elastic modulus of PA6 material, tensile samples, as illustrated in Figure 1, were designed. The Young’s modulus of the lattice material was measured at ambient temperatures of 23 °C (room temperature), 50 °C, and 100 °C. Experimental results demonstrated that at room temperature, the material’s Young’s modulus was 934 MPa (E1). This value decreased to 564 MPa (E2) at 50 °C and plummeted to 200 MPa (E3) at 100 °C, indicating a significant softening behavior under elevated temperatures.

### 2.2. Structural Design

In this study, the cement-based composite specimen is a cube with each side measuring 42 mm, resulting in a total volume of 74,088 mm^3^. Following the guidelines provided in the literature [21], this paper explores six distinct 3D-printed polymer lattice configurations for reinforcement. The selected cellular structures are circular, cubic, Kelvin, octagonal (Oct), rhombicuboctahedron (RO), and a reinforced octagon variant (SO). Each 3D-printed lattice is designed to occupy a volume fraction of 8% within the cement matrix (Table 4). The diameters of the lattice cells, varying according to their structural forms, are determined using the formula provided below:

(1) $$V_{circular}=f(d_{1})=98.125d_{1}+4240.9d_{1}^{2}-400.94d_{1}^{3}$$

(2) $$V_{cubic}=f(d_{2})=0.0005d_{2}+2356.2d_{2}^{2}-121.52d_{2}^{3}$$

(3) $$V_{kelvin}=f(d_{3})=0.00005d_{3}+4804.4d_{3}^{2}-497.67d_{3}^{3}$$

(4) $$V_{Oct}=f(d_{4})=910.78d_{4}+4045.2d_{4}^{2}-31.93d_{4}^{3}$$

(5) $$V_{RO}=f(d_{5})=-2\times 10^{-5}d_{5}+8115.9d_{5}^{2}-1151.5d_{5}^{3}$$

(6) $$V_{so}=f(d_{6})=0.0003d_{6}+7072.3d_{6}^{2}-900.55d_{6}^{3}$$

In the numerical simulation, the polymer lattice is integrated within the cement matrix, serving as an internal constraint condition. This configuration ensures that the lattice is encased by the cement matrix, effectively simulating its embedded position.

In conducting numerical simulations with ABAQUS (2022) software, aligning closely with physical laboratory operations improves the accuracy of the simulations [24,25,26]. This involves designing rigid plates to interact with both the upper and lower surfaces of the cube specimen, thereby restricting its movement. This simulation step is critical for accurately replicating the compressive constraints applied by the laboratory’s compression apparatus on the specimen. For the rigidity-enforced plates, it is crucial to apply fixed constraints to the lower surface to simulate the restraining effect of the laboratory compressor’s heads. This entails limiting displacement in all directions, effectively preventing any movement of the surface. In ABAQUS, such a scenario is facilitated through the application of boundary conditions that enforce these fixed constraints. Conversely, the plate on the upper surface is subjected to a fixed displacement constraint, which allows for the specification of displacement in certain directions while permitting the application of force or displacement in others. Given the focus on uniaxial compression tests in this simulation, a vertical displacement constraint is applied perpendicular to the sample surface. This approach effectively simulates the compressive force exerted during the actual laboratory test.

### 2.3. Finite Element Simulation 

ABAQUS, a finite element simulation software, is extensively utilized across various engineering disciplines due to its robust capabilities. It facilitates a broad spectrum of analyses, ranging from basic linear to intricate nonlinear problems, enabling the calculation and analysis of the mechanical behavior of complex engineering structures with high precision. This study employs ABAQUS to conduct a numerical simulation of a uniaxial compression experiment on cement-based composites, specifically focusing on specimens without any pre-existing cracks.

**Figure 2:**
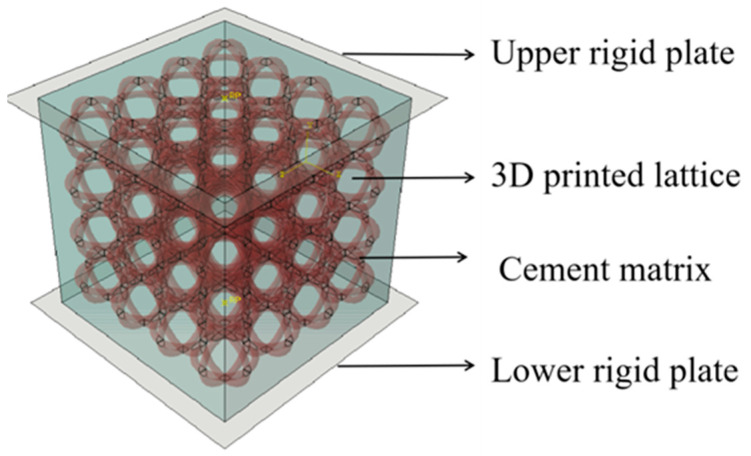
ABAQUS computational modeling.

Figure 2 illustrates the schematic representation of the finite element model used in the simulation. The process of conducting simulations using ABAQUS (2022) software encompasses several key steps, detailed as follows:

Firstly, a geometric model is established through 3D modeling of the polymer lattice within the sample, utilizing CAD (2023) software. This model is then exported and imported into ABAQUS, where the component is constructed as a cohesive entity within the assembly module.

The analysis step size is adjusted by configuring the output variables for each step of the analysis. 

Regarding interactions, within a specific region and scope, interactions and mutual constraints exist between models. Specifically, contact is established between the upper and lower rigid plates and the corresponding upper and lower surfaces of the sample. Furthermore, the 3D-printed lattice introduces internal constraints within the cement matrix, as depicted in the figure.

Mesh discretization involves dividing the entity into grids. Given the complexity of the 3D-printed lattice structure, alongside its small cellular dimensions, the mesh is configured as tetrahedral. The mesh size is finely adjusted based on the varying structural forms to optimize computational outcomes. In contrast, the cement matrix exhibits a regular cubic structure, which is discretized using a hexahedral mesh.

The loading methodology involves establishing a reference point on the rigid plate located at the model’s upper surface. A vertical downward load is applied at this reference point to simulate the pressure dynamics observed in uniaxial compression testing. In this study, a fixed displacement loading approach was employed, with the displacement set at 5% of the sample’s side length, to apply the load to the sample.

## 3. Results and Discussion

This study examined six varieties of cement-based composites reinforced with 3D-printed polymer lattices of different structural configurations through uniaxial compression numerical simulations. By varying the elastic modulus of the polymer materials, the simulation explored how different ambient temperature conditions affect the compressive strength of these 3D-printed polymer lattice-reinforced cement-based composites. The mechanical and deformation properties of the samples were determined by comparing stress–strain curve analyses and strain distribution maps with the results obtained from ABAQUS numerical simulations.

### 3.1. Stress–Strain Curve Analysis

**Figure 3:**
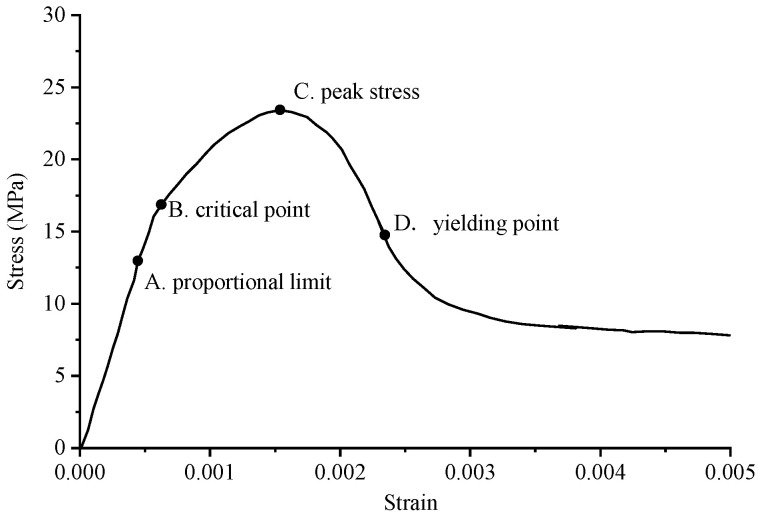
The stress–strain curve of the standard concrete specimen under uniaxial compression test.

**Figure 4:**
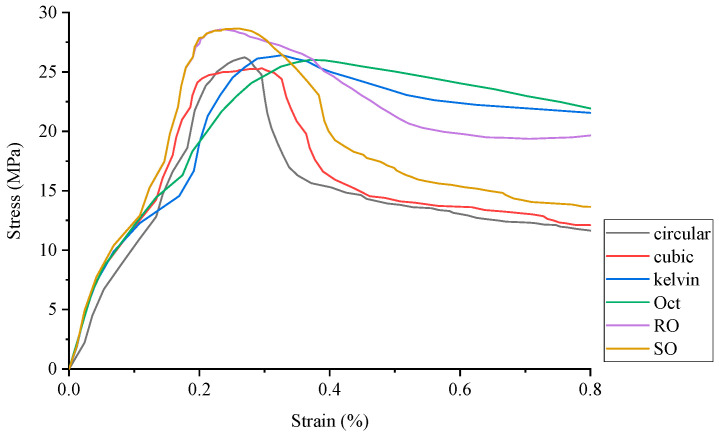
Numerical simulation of stress–strain curves for uniaxial compression of specimens strengthened with different lattice structures.

**Figure 5:**
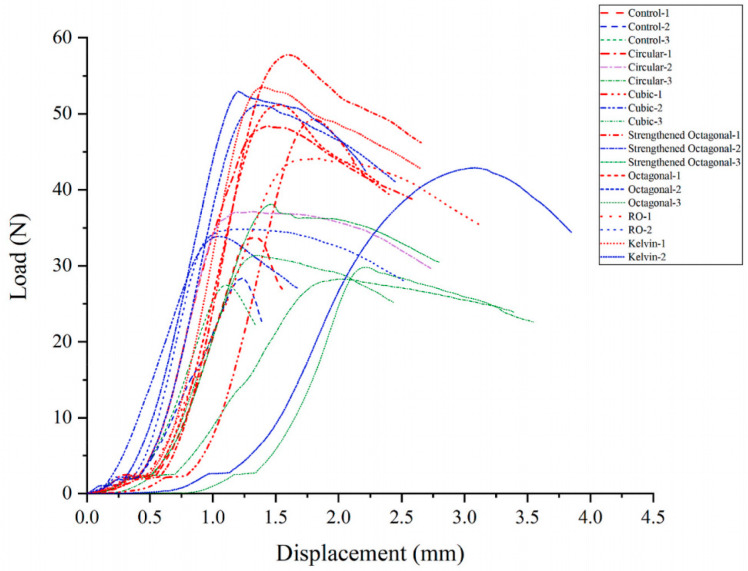
Laboratory-displacement curve for compressive test of cement-based specimens.

The stress–strain curve of conventional concrete material is shown in Figure 3, and the numerical simulation results and experimental results of uniaxial compression are shown in Figure 4 and Figure 5, respectively. It can be observed that the stress–strain curve of the 3D printed lattice-reinforced cement-based composite is consistent with that of conventional concrete materials. By comparing the simulation results with the experimental results, it is shown that the compressive mechanical properties of cement-based materials with different mesh structures are similar under uniaxial compression. These findings delineate the mechanical response process of 3D-printed polymer lattice-reinforced cement-based composite specimens under uniaxial compression, characterized by the following stages:

(1) Initial Stage (O-A): During this phase, the stress level reaches approximately 70% to 85% of the peak stress. At this point, the deformation observed in the specimen is predominantly elastic, resulting from the interaction between the cement matrix and the polymer lattice. This behavior is interpreted as linear elastic deformation, evident from the near-linear relationship depicted in the stress–strain curve. Concurrently, material displacement changes are minimal, despite the significant alterations in the load.
(2) Second Stage (A-B): At this stage, stress levels range from approximately 85% to 93% of the peak stress. The deformation behavior of the specimen is marked by the gradual emergence and expansion of small cracks within the sample, commensurate with the loading process.
(3) Second Stage (A-B): In this stage, the stress reaches roughly 85% to 93% of the peak load. The stage is characterized by the gradual appearance and steady expansion of small cracks within the specimen.
(4) Third Stage (B-C): Stress levels during this stage approximate 93% to 100% of peak stress. This phase is marked by a deceleration in the material’s compressive capacity enhancement. Concurrently, the stress–strain curves exhibit an increasing curvature, transitioning towards a more gradual trend. The predominant deformation observed in the specimen is of an irreversible plastic nature, with a minor component of elastic deformation also present at this stage.
(5) Fourth Stage (C-): Upon exceeding the peak stress, the sample’s compressive properties begin to diminish correspondingly. As the external force applied to the material escalates with continued loading, the damage to the specimen progressively worsens.

Additionally, the maximum stress the sample can endure before failure is designated as the peak stress, depicted at point C in Figure 4. The stress–strain curve of concrete material serves as a metric for assessing the effectiveness of cement-based composites reinforced by 3D-printed polymer lattice structures. Notably, 85% of the peak stress is utilized as the demarcation between the plastic deformation and elastic deformation phases, corresponding to point B in Figure 3.

An analysis of Table 2, which presents peak stress data from the numerical simulations of different lattice structures, reveals a consistent trend: a decrease in the elastic modulus of the 3D-printed lattice results in a reduction in the peak stress experienced by all specimens compared to the control specimens. Specifically, in Table 5 when the lattice’s elastic modulus decreases to 60.4% of the control modulus, the peak load of the cement-based composite reinforced with a Circular lattice is 0.8% lower than that of the ideal condition. With the use of Cubic, Kelvin, Oct, RO, and SO lattice configurations as reinforcements, the peak loads decrease by 0.8%, 0.14%, 17.82%, 10.57% and 11.73%, respectively.

**Figure 6:**
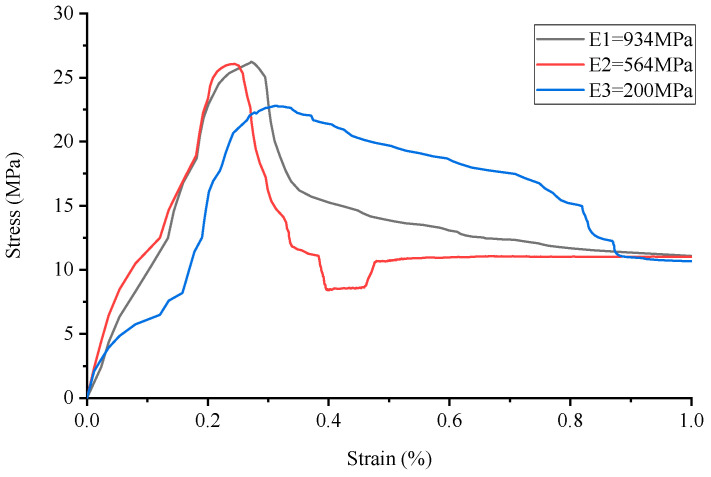
Stress–strain curve of Circular lattice reinforced cement-based composites.

The analysis of the stress–strain curve for 3D-printed Circular lattice reinforced cement-based composite materials, as illustrated in Figure 6 reveals that the contribution of the 3D-printed polymer lattice to the overall cement-based material is minimal, with its volume fraction being only 8%. Consequently, the reduction in its elastic modulus exerts a negligible impact on the peak stress of the cement-based composite samples. Nevertheless, following the failure of the cement matrix, the influence of elastic modulus attenuation on the peak stress of the cement-based composite specimens becomes marginally more significant. It is observed that during the failure stage, denoted as the C-stage, the reduction in the elastic modulus of the 3D-printed lattice has a more pronounced effect on the compressive strength of the sample.

For instance, when the elastic modulus is set at 564 MPa, there is no significant decrease in the peak stress of the sample. However, following the degradation of the cement matrix, a notable trend is observed during the failure stage (referred to as the C-stage); the reduction in the elastic modulus due to environmental temperature fluctuations has a marked impact on the compressive strength of the sample. Conversely, at an elastic modulus of 200 MPa, analysis of the corresponding figure demonstrates a discernible decrease in the peak stress of the material under these experimental conditions.

**Figure 7:**
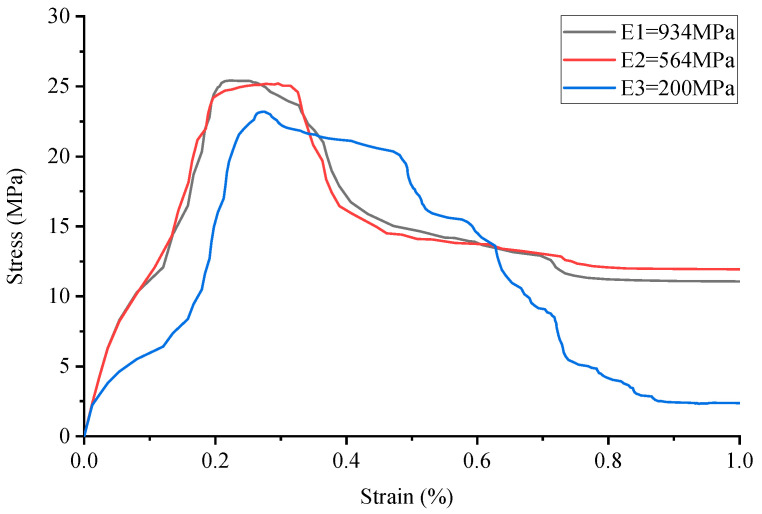
Stress–strain curve of Cubic lattice reinforced cement-based composites.

**Figure 8:**
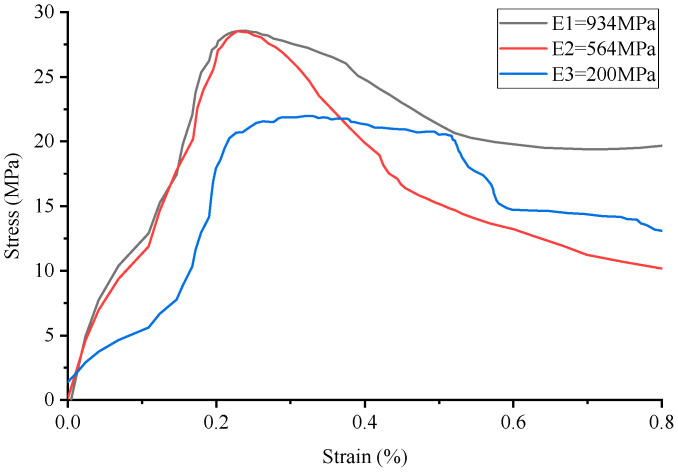
Stress–strain curve of Kelvin lattice reinforced cement-based composites.

**Figure 9:**
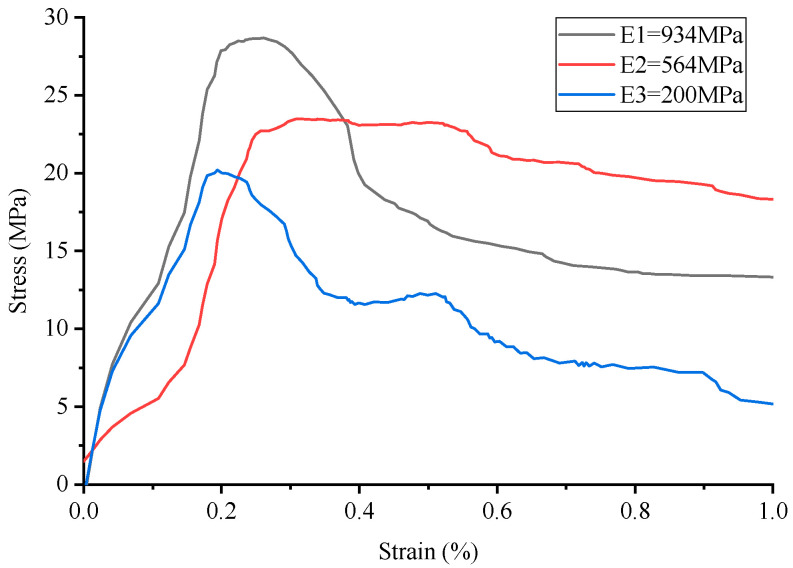
Stress–strain curve of Oct lattice reinforced cement-based composites.

**Figure 10:**
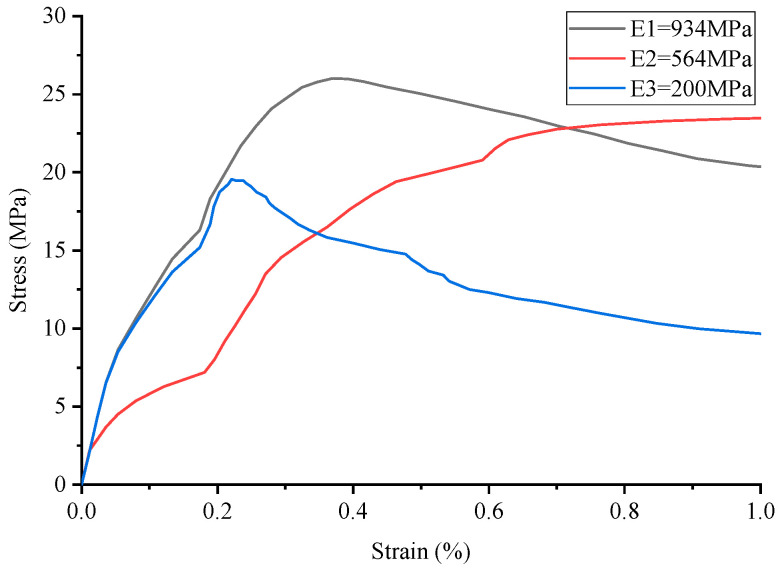
Stress–strain curve of RO lattice reinforced cement-based composites.

**Figure 11:**
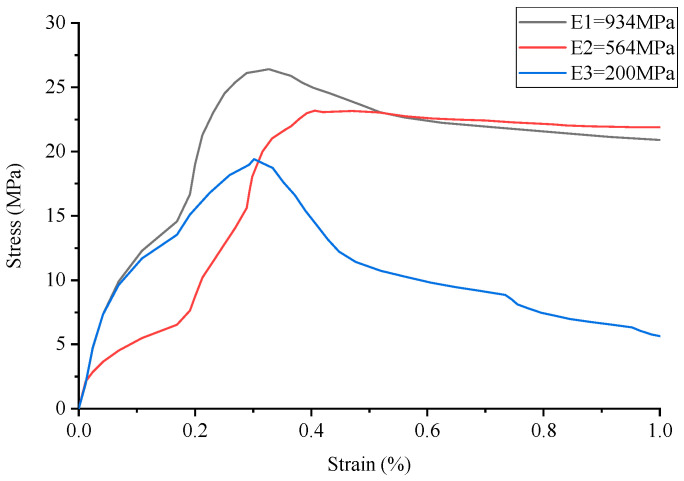
Stress–strain curve of SO lattice reinforced cement-based composites.

By comparing the numerical simulation outcomes for five distinct structural configurations, as depicted in Figure 6, Figure 7, Figure 8, Figure 9, Figure 10 and Figure 11, it becomes evident that variations in ambient temperature lead to a diminution of Young’s modulus in the 3D-printed polymer lattice. This reduction directly contributes to a decline in the peak stress of the cement-based material, subsequently impairing its performance.

### 3.2. Strain Analysis

In the uniaxial compression experiment, as load is applied, the specimen experiences cracking, leading to its failure. The physical test phase employed the DIC monitoring technique to examine the deformation and the areas of surface cracking under compression. This study simulated the stress–strain relationship of the specimen prior to cracking in a uniaxial compression setting using finite element simulation. Accordingly, this chapter analyzes the strain distribution patterns in the specimen to forecast potential failure points.

**Figure 12:**
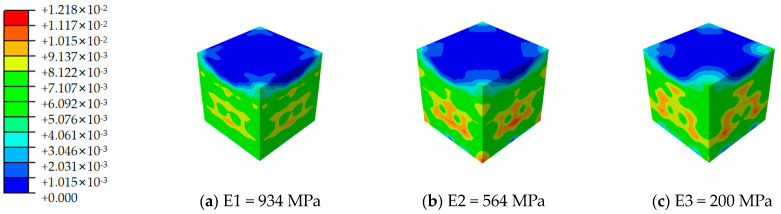
Strain cloud image of cement-based samples reinforced with circular lattice structures at different ambient temperatures.

**Figure 13:**
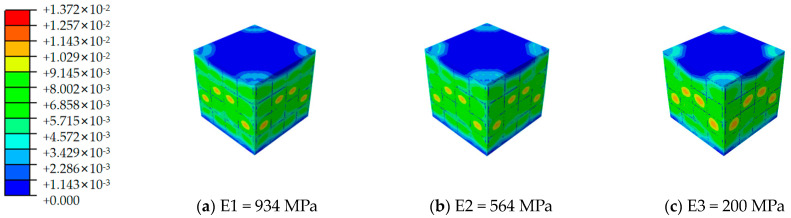
Strain cloud image of cement-based samples reinforced with cubic lattice structures at different ambient temperatures.

**Figure 14:**
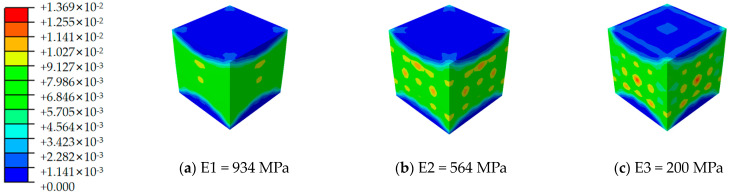
Strain cloud image of cement-based samples reinforced with kelvin lattice structures at different ambient temperatures.

**Figure 15:**
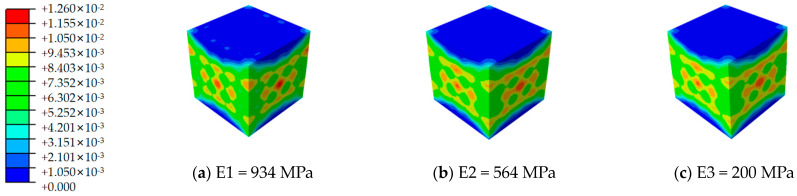
Strain cloud image of cement-based samples reinforced with Oct lattice structures at different ambient temperatures.

**Figure 16:**
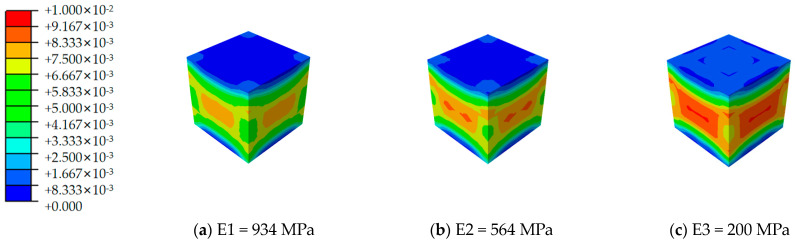
Strain cloud image of cement-based samples reinforced with RO lattice structures at different ambient temperatures.

**Figure 17:**
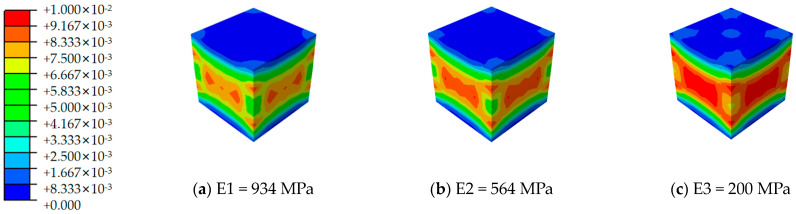
Strain cloud image of cement-based samples reinforced with SO lattice structures at different ambient temperatures.

Figure 12, Figure 13, Figure 14, Figure 15, Figure 16 and Figure 17 depict the stress–strain curves for six varieties of cement-based composites reinforced with 3D-printed lattices, derived from numerical simulations of uniaxial compression. Specifically, Figure 12 illustrates the evolution of strain distribution in cement-based composites reinforced with circular lattice specimens under varying ambient temperatures. As the ambient temperature increases, Young’s modulus of the polymer lattice diminishes, leading to a reduction in strain capacity and an enlargement of the strain distribution region. Notably, when the ambient temperature reaches 100 °C and Young’s modulus of the polymer decreases to 200 MPa, there is a marked increase in the specimen’s propensity for failure under pressure. This trend suggests that at elevated temperatures, the compressive properties of the polymer lattice are significantly compromised. Coupled with the cracking of the cement matrix, the lattice becomes increasingly susceptible to ambient temperature effects, heightening the likelihood of specimen failure. This observation underscores the fact that high-temperature environments substantially degrade the stability and load-bearing capabilities of lattice-reinforced cement-based materials, elevating the risk of material failure.

For cement matrix composites, the stress–strain curves under vertical compression can generally be segmented into two phases: the ascending and descending phases. In the initial compression phase, the exerted load on the specimen is minimal, and the corresponding deformation primarily results from the elastic deformation of the material’s internal structure, rendering the stress–strain curve nearly linear. At this juncture, the specimen predominantly exhibits compression zones. Strain distribution analyses reveal that the cement base test block is subjected to relatively low pressure without significant stress concentration zones. This suggests a fairly uniform stress distribution throughout the material during the initial loading phase, indicating an absence of marked stress concentration.

As the loading process progresses, the stress–strain curve transitions into a descending phase where the material begins to exhibit plastic deformation and the impact of stress concentration on the specimen becomes increasingly evident. In high-temperature conditions, the thermal expansion of the material’s various phases and the reduction in Young’s modulus of the polymer lattice contribute to diminished compressive performance, leading to a more uneven strain distribution and elevating the risk of material damage. The alterations observed in the strain distribution maps from numerical simulations further elucidate the deterioration in compressive mechanical properties of cement-based composites reinforced with 3D-printed lattices when subjected to high temperatures. These simulations provide insights into the failure dynamics and mechanical property degradation of materials across different ambient temperature conditions.

## 4. Conclusions

In this study, ABAQUS (2022) software facilitated the numerical simulation of uniaxial compression tests on composite materials featuring 3D-printed polymer lattices embedded within a cement matrix. This approach yielded a series of insightful conclusions, which are instrumental in elucidating the compressive mechanical properties of cement-based composites under varying high-temperature conditions. Presented below is an analytical summary of the key research findings:

(1) Through the creation of a precise finite element model and the simulation of laboratory uniaxial compression tests, this study has successfully validated the accuracy of its numerical analysis model. This achievement not only furnishes a dependable simulation methodology for subsequent research endeavors but also lays a robust groundwork for the enhanced analysis and application of experimental data. Moreover, the process of developing and validating the model serves as a valuable benchmark for the numerical simulation of analogous materials and structures.
(2) The study reveals that variations in ambient temperature markedly influence the elastic modulus of 3D-printed polymer materials, subsequently altering the compressive mechanical properties of cement-based composites. This finding underscores the necessity of accounting for material performance shifts under diverse temperature conditions in practical engineering applications to ensure the reliability and safety of structures.
(3) This study demonstrates that the compressive strength of composite materials tends to decrease as the elastic modulus of polymer materials is reduced. This observation holds significant implications for the optimization of composite material design and the enhancement of their structural characteristics.
(4) The findings indicate that as Young’s modulus of the polymer decreases, the strain region widens while the maximum strain diminishes, suggesting an impact on both the ductility and load-bearing capacity of the structure. Furthermore, when the elastic modulus falls to a specific critical threshold, specimen cracking occurs at the onset of the compression test. This highlights the necessity for meticulous attention to the lower limits of material properties during the design process to prevent premature failure.

In conclusion, this study not only introduces a novel approach for examining the mechanical properties of cement-based composites but also lays a crucial theoretical and practical foundation for material design and structural optimization in related domains. Building upon this foundation, future investigations can delve into the properties of various material combinations, structural configurations, and loading scenarios, thereby broadening the application spectrum of cement-based composites in diverse fields.

## Figures and Tables

**Table 1:** 3D-printing polymer material parameters.

**Density (kg/m** **^3^)**	2.4 × 10^−9^
**Young’s modulus (GPa)**	30
**Poisson’s ratio**	0.2
**expansion angle (°)**	30
**eccentricity ratio**	0.1
**fb0/fco**	1.16
**k**	0.6667
**Viscous parameter**	0.0005

**Table 2:** PA6 material parameters.

Density (kg/m^3^)	Indoor Temperature Young’s Modulus (MPa)	Poisson’s Ratio
1.1 × 10^−9^	934	0.38

**Table 3:** Elastic modulus of PA6 at different ambient temperatures.

Environment Temperature	Indoor Temperature	50 °C	100 °C
elasticity modulus	934 MPa	564 MPa	200 MPa

**Table 4:** 3D-printed lattice structures.

Structure Serial Number	Lattice Unit Cell Type	Unit Cell Design	Cell Design Parameters (mm)	CAD Southwest View
1	circle	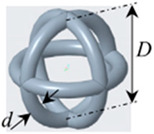	D = 10, d = 1.246	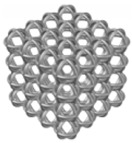
2	cubic	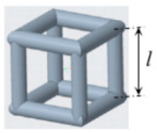	l = 10, d = 1.659	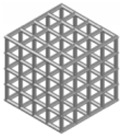
3	kelvin	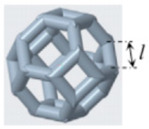	l = 3.54, d = 1.183	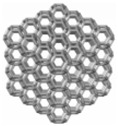
4	octagonal (Oct),	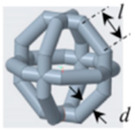	l = 4.14, d = 0.979	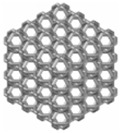
5	rhombicubactahedron (RO)	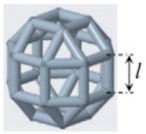	l = 1.414, d = 0.916	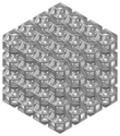
6	strengthened octagon octagonal (SO)	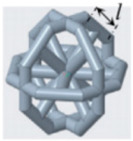	l = 3.54, d = 0.979	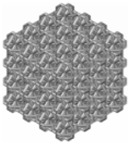

**Table 5:** Peak stress of lattice numerical simulation of different structures.

	Elasticity Modulus	E_1_ = 934 MPa	E_2_ = 564 MPa	E_3_ = 200 MPa
Crystal Structure				
Circular		26.23	26.04	22.74
Cubic		25.23	25.17	23.16
Kelvin		28.49	28.45	21.79
Oct.		28.57	23.48	20.21
RO		26.02	23.27	19.46
SO		26.26	23.18	19.41

## Data Availability

Data are contained within the article.

## References

[1] Carpinteri A. (2012). Mechanical Damage and Crack Growth in Concrete: Plastic Collapse to Brittle Fracture.

[2] Hrițuc A., Mihalache M.A., Dodun O., Nagîț G., Beșliu-Băncescu I., Rădulescu B., Slătineanu L. (2023). Propagation of Sounds through Small Panels Made of Polymer Materials by 3D Printing. Polymers.

[3] Khosravani M.R., Ayatollahi M.R., Reinicke T. (2023). Effects of post-processing techniques on the mechanical characterization of additively manufactured parts. J. Manuf. Process..

[4] Johnson L.K., Richburg C., Lew M., Ledoux W.R., Aubin P.M., Rombokas E. (2018). 3D Printed lattice microstructures to mimic soft biological materials. Bioinspir. Biomim..

[5] Kang J.D., Kim S. (2021). Development of a 3D printing method for the textile hybrid structure. Int. J. Cloth. Sci. Technol..

[6] Park S., Shou W., Makatura L., Matusik W., Fu K. (2022). 3D printing of polymer composites: Materials, processes, and applications. Matter.

[7] Xu Y., Zhang H., Šavija B., Figueiredo S.C., Schlangen E. (2019). Deformation and fracture of 3D printed disordered lattice materials: Experiments and modeling. Mater. Des..

[8] Qin S., Cao S., Yilmaz E. (2022). Employing U-shaped 3D printed polymer to improve flexural properties of cementitious tailings backfills. Constr. Build. Mater..

[9] Balla V.K., Kate K.H., Satyavolu J., Singh P., Tadimeti J.G.D. (2019). Additive manufacturing of natural fiber reinforced polymer composites: Processing and prospects. Compos. Part B Eng..

[10] Nam Y.J., Hwang Y.K., Park J.W., Lim Y.M. (2019). Feasibility study to control fiber distribution for enhancement of composite properties via three-dimensional printing. Mech. Adv. Mater. Struct..

[11] Farina I., Fabbrocino F., Carpentieri G., Modano M., Amendola A., Goodall R., Feo L., Fraternali F. (2016). On the reinforcement of cement mortars through 3D printed polymeric and metallic fibers. Compos. Part B Eng..

[12] Tian H., Zhou Z., Zhang Y., Wei Y. (2020). Axial behavior of reinforced concrete column with ultra-high performance concrete stay-in-place formwork. Eng. Struct..

[13] Rosewitz J.A., Choshali H.A., Rahbar N. (2019). Bioinspired design of architected cement-polymer composites. Cem. Concr. Compos..

[14] Xu Y., Šavija B. (2019). Development of strain hardening cementitious composite (SHCC) reinforced with 3D printed polymeric reinforcement: Mechanical properties. Compos. Part B Eng..

[15] Salazar B., Aghdasi P., Williams I.D., Ostertag C.P., Taylor H.K. (2020). Polymer lattice-reinforcement for enhancing ductility of concrete. Mater. Des..

[16] Ding S., Xiang Y., Ni Y.Q., Thakur V.K., Wang X., Han B., Ou J. (2022). In-situ synthesizing carbon nanotubes on cement to develop self-sensing cementitious composites for smart high-speed rail infrastructures. Nano Today.

[17] Han B., Sun S., Ding S., Zhang L., Yu X., Ou J. (2015). Review of nanocarbon-engineered multifunctional cementitious composites. Compos. Part A Appl. Sci. Manuf..

[18] Ráž K., Chval Z., Thomann S. (2023). Minimizing Deformations during HP MJF 3D Printing. Materials.

[19] Robar J.L., Kammerzell B., Hulick K., Kaiser P., Young C., Verzwyvelt V., Cheng X., Shepherd M., Orbovic R., Fedullo S. (2022). Novel multi jet fusion 3D-printed patient immobilization for radiation therapy. J. Appl. Clin. Med. Phys..

[20] Shalchy F. (2016). An Investigation on Interfacial Adhesion Energy between Polymeric and Cellulose-Based Additives Embedded in CSH Gel. Master’s Thesis.

[21] Kakavand M.R., Taciroglu E., Hofstetter G. (2020). Evaluation of the Performance of an Enhanced Damage Plasticity Model for Predicting the Cyclic Response of Plain Concrete under Multiaxial Loading Conditions. Int. J. Struct. Civ. Eng. Res..

[22] Voyiadjis G.Z., Taqieddin Z.N. (2009). Elastic plastic and damage model for concrete materials: Part I-theoretical formulation. Int. J. Struct. Chang. Solids.

[23] Lee J., Fenves G.L. (1998). Plastic-damage model for cyclic loading of concrete structures. J. Eng. Mech..

[24] Jiang L., Zhao C., Zhang J. (2021). Calculation and Analysis of Sluice Bottom Seepage with Two Methods. IOP Conf. Ser. Earth Environ. Sci..

[25] Merzuki M.N.M., Ma Q., Rejab M.R.M., Sani M., Zhang B. (2022). Experimental and numerical investigation of fibre-metal-laminates (FMLs) under free vibration analysis. Mater. Today Proc..

[26] Rizwan M., Liang Q.Q., Hadi M.N.S. (2021). Fiber-based computational modeling of rectangular double-skin concrete-filled steel tubular short columns including local buckling. Eng. Struct..

